# Seroconversion and Skin Mucosal Parameters during Koi Herpesvirus Shedding in Common Carp, *Cyprinus carpio*

**DOI:** 10.3390/ijms21228482

**Published:** 2020-11-11

**Authors:** Irene Cano, Brian Mulhearn, Sabiha Akter, Richard Paley

**Affiliations:** International Centre of Excellence for Aquatic Animal Health, Cefas Weymouth Laboratory, Weymouth, Dorset DT4 8UB, UK; brian.mulhearn@icloud.com (B.M.); sabiha031@gmail.com (S.A.); richard.paley@cefas.co.uk (R.P.)

**Keywords:** koi herpesvirus, viral shedding, seroconversion, lysozyme, complement, IgM, IgZ2, mucosal immunity, skin swabs, common carp

## Abstract

Seroconversion and the mucosal *lysozyme G* (*lysG*), *complement 3* (*c3*), and *immunoglobulins M* (*IgMsec*) and *Z2* (*IgZ2*) were measured for up to 900 degree days (DD) in skin swabs from common carp exposed to koi herpesvirus (KHV or CyHV-3) at either a non-permissive temperature (12 °C) or permissive temperatures (17 and 22 °C), and in survivors subjected to temperature increase to 22 °C 500 DD after the initial exposure. The survival rate at 22 °C varied from 100% in fish initially exposed at 12 °C, to 20% at 17 °C and 0% at 22 °C. Viral shedding episodes lasted for up to 29 days (493 DD) for fish clinically infected at 17 °C, and up to 57 days (684 DD) for asymptomatic fish held at 12 °C. Up-regulation of *lysG* transcripts was measured at 17 and 22 °C. Down-regulation of *c3* and *IgMsec* transcripts was measured independent of the water temperature, followed by up-regulation after the temperature increase coinciding with seroconversion and clearance of KHV from the skin mucus. *IgZ2* mRNA showed a negative correlation with *IgM* transcripts. KHV subversion of the complement system at the mucosal site coupled with poor immunoglobulin secretion during the viral replication might contribute to the long window of viral shedding, thus facilitating viral transmission.

## 1. Introduction

Koi herpesvirus disease (KHVD) emerged as a problem in recreational fisheries of common carp (*Crypinus carpio* Linnaeus, 1758) in the United Kingdom (UK) in 2003. First described as affecting ornamental carp (koi carp) in Europe, Israel, and the United States in the 1990s [1], it was soon observed worldwide (except for Australia) due to the intense global trade of koi and common carp [2]. Since 2006, KHVD became a reportable disease to the OIE (Office International des Epizooties) and subsequently was made notifiable in the UK in 2007 with the implementation of the European Directive 2006/88/EC [3].

The causative agent of KHVD, the koi herpesvirus (KHV) or Cyprinid herpesvirus 3 (CyHV-3), is a large enveloped double-stranded DNA virus belonging to the *Alloherpesviridae* family [4]. KHV primarily infects epithelial cells of skin and gills, after which it becomes viraemic, causing a systemic infection, finally infecting cells of the central nervous system [5,6]. KHVD clinical signs manifest as irregular patches on the skin and gills and severe gill necrosis. Typically, clinically infected fish show appetite loss, increased respiratory frequency, and erratic swimming before death [7]. Excess mucus production is noticeable at the early stages of the infection coinciding with high loads of virus shedding throughout the skin [8]. Mortalities occur soon after the infection due to severe osmotic shock as a consequence of the sloughing of the skin, with late deaths associated with severe gill necrosis and encephalitis around 3 weeks after infection [6].

KHVD outbreaks are highly influenced by water temperature. The likelihood of transmission and development of viremia typically occurs when the water temperature is between 16–25 °C, with an optimal temperature between 22–24 °C [9]. At these temperatures, death occurs within 24 to 48 h after the initial onset of clinical signs, causing mortality rates of ~90% of the infected population [10]. Infection at temperatures lower than 16 °C often results in asymptomatic infection and reduced pathology, with no mortalities recorded below 13 °C [7]. Despite mathematical models and field data suggesting that KHVD outbreaks could be controlled by careful management of water temperature in an aquaculture setup [11], studies on KHV-host response at non-permissive temperatures are scarce.

As described for other herpesviruses [12], KHV establishes a lifelong latent infection. It has been demonstrated that peripheral blood leukocytes and other tissues (i.e., neurons) support KHV latency in carp [13,14], and that KHV can, asymptomatically or symptomatically, reactivate from the latency stage by temperature stress between 17–23 °C [14,15], which can lead to sporadic shedding episodes and outbreaks [16], subsequently sustaining high KHV-antibody titers on the population over time [17].

Recent studies on the mucosal immune system of teleost fish have highlighted an important role of the skin, gills, and gut immune cells and molecules in response to pathogens. Teleost mucosal surfaces contain mucus-producing cells similar to the mammalian type I mucosal surface [18], a plethora of hematopoietic cells (T cells, macrophages, mast cells, and dendritic cells, for review see [19]), a specific immunoglobulin (Ig) to mucosal surfaces (IgT/IgZ) [20], the polymeric Ig receptor (pIgR) expressed by epithelial cells in gut and skin [21], and its secretory component with associates with IgM and IgT/IgZ mucus [21].

Fish skin mucus contains many humoral non-specific defence factors such as lysozyme, complement, interferon (IFN), C-reactive protein, lectin (haemagglutinin), haemolysin and transferrin, and other antimicrobial peptides mainly secreted by goblet cells [22,23]. The antimicrobial lysozyme is a mucolytic enzyme highly expressed in neutrophils, macrophages, and granulocytes. The specific antiviral function of lysozyme has been shown in humans [24,25], in fish its expression activates the complement system and phagocytes [26]. In common carp, both the c- and g-type lysozyme [27,28], as well as a positive correlation of lysozyme activity with antigen-antibody serum titre [29], have been reported. In teleost fish, lysozyme in serum is highly modulated by the water temperature, showing decreased levels at a lower temperature [30]. Similar to mammals, the complement activation pathways (classical, lectin, and alternative pathway), as well as the cytolytic pathway are present in teleosts [31,32]. However, despite the importance of complement on mucosal barriers, the number of studies addressing the role of the complement in fish skin in pathogen infections is reduced [33].

Studies on the immune response to KHV are limited. At permissive temperatures for disease (acute phase of the infection), a reduction in the gene expression of important components of the mucosal barrier of the skin, in particular *mucin 5B*, *beta-defensin 1* and *2*, and the tight junction proteins *claudin 23* and *30* were reported [34]. Differential immune responses have been observed between KHV-resistant and susceptible carp lines, in particular, a higher expression of immune-related genes, involving pathogen recognition, complement activation, MHC class I-restricted antigen presentation, and development of adaptive mucosal immunity [35,36] were seen in resistant lines. In the spleen of infected common carp, the host *IFNy* and interleukin (*il)-10*, as well as the viral *interleukin* (*il*)*10* homologue, were shown to be consistently up-regulated in different phases of the KHV infection [37].

Though these studies provide important information, very little is known about the role of mucosal immunity and humoral responses during subclinical infections. The present study compared the gene expression of key mucosal parameters during KHV infection at permissive (clinical infection) and non-permissive (subclinical infection) temperatures, after which the animals were subjected to temperature increase (heat stress to 22 °C) to study the role of the seroconversion on the viral reactivation.

## 2. Results

### 2.1. Exposure at Lower Temperatures Increased Survival

All common carp exposed to KHV at 22 °C showed a fast progression of the infection. Skin darkening and mucus production were observed from day 2 (44 DD) after the challenge in all the animals, sloughing of the epidermis followed by moribundity was observed in 90% (9/10) by day 8 (176 DD), and by day 12 (264 DD) in the remaining individual.

Fish exposed to KHV at 17 °C showed a slow progression of typical KHVD clinical signs, consisting of a reduced feeding response and petechial haemorrhages in 60% of the animals at day 4 (68 DD), which progressed to ulcerative dermal wounds, darkening of the skin and severe mucus production at day 10 (170 DD). Those fish died between day 10 and 27 (459 DD). The remaining fish (40%) exposed to an increase of temperature up to 22 °C (heat stress) at 500 DD, showed severe lethargy and inappetence, with a total of 80% cumulative mortality at the end of the challenge.

**Figure 1:**
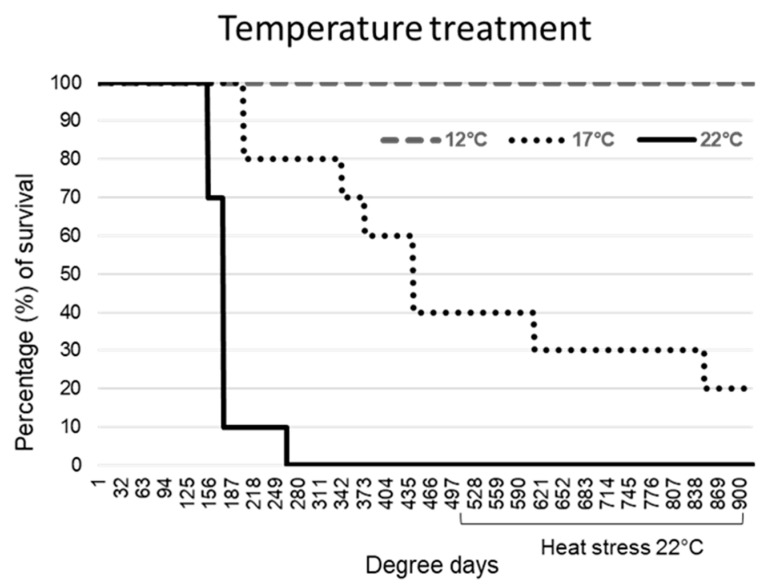
Survival curve of common carp infected with koi herpesvirus (KHV) at 12 °C, 17 °C or 22 °C. At ~500 degree days (29 days for fish at 17 °C and 42 days at 12 °C), all survivors were subjected to temperature raise to 22 °C. *n* = 10 per group.

All fish bath exposed to KHV at 12 °C survived the primary infection and the posterior heat stress (Figure 1). A reduced feeding response was noted during the first week after the challenge. Mild skin lesions consisting of closed skin ulcerations and mild diffuse petechial epidermal haemorrhages were observed from day 5 (60 DD) to 36 (432 DD) in 60% of the fish, resolving by the healing of the wound. After the heat stress, a mild excess of mucus production was observed in 30% of the fish from day 50 (600 DD) to 57 (684 DD) with no other clinical signs observed. No signs of KHVD or stress were observed in control fish associated with temperature changes or with the repetitive skin swabbing.

### 2.2. Heat Stress Boosted Antibody Levels in Sera

**Figure 2:**
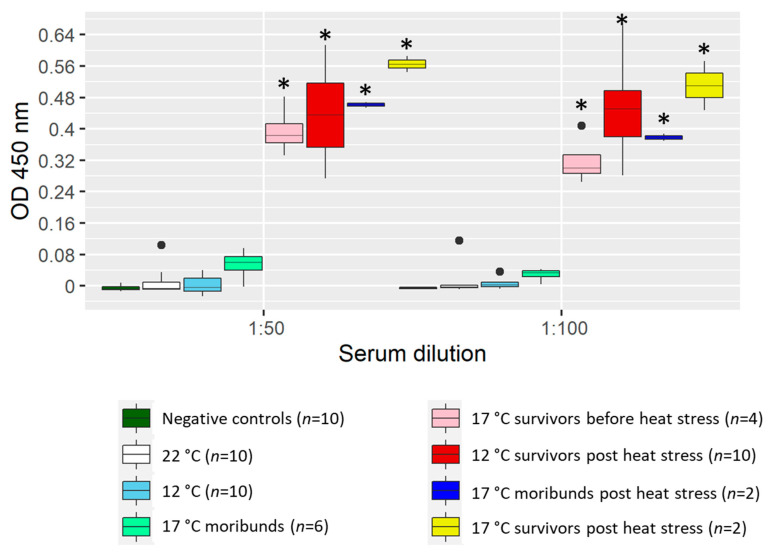
ELISA detection of koi herpesvirus (KHV) antibodies in serum from challenged common carp before and after an increase of temperature to 22 °C (heat stress). Sera were tested at 1:50 and 1:100 dilutions. Each bar represents from each temperature and fish group the median (cross), upper and lower quartile (box), and upper and lower extreme (line) of the optical density (OD) at 450 nm. Single points indicate outliers. Asterisks denote significant differences (Student’s *t*-test *p*-value < 0.05) between KHV-infected and control groups.

All fish challenged at 22 °C, which succumbed rapidly to the disease (<500 DD), did not develop detectable anti-KHV antibody levels in serum (Figure 2).

Similarly, fish at 17 °C that died before 500 DD (6 out of 10 fish) did no show significant levels of anti-KHV antibody in the ELISA test when compared with control fish. The rest of the fish at 17 °C that survived more than 500 DD tested positive for KHV antibodies in sera.

Fish held at 12 °C did not develop measurable anti-KHV antibodies before the heat stress (<500 DD), however, high levels of antibodies were measured after the heat stress in surviving fish sampled at ~900 DD. There were no detectable levels of KHV antibody in sera of specific pathogen-free (SPF) control fish.

### 2.3. Suitability of Non-Invasive Skin Swabs for Gene Expression Analysis

RNA extractions from a total of 390 non-lethal skin swabs yielded concentrations of RNA between 2–330 ng µL^−1^, with an average of 21 ± 23 ng µL^−1^ in asymptomatic fish and 145 ± 58 ng µL^−1^ in those showing clinical signs of KHVD. The qPCR cycle quantification (Ct) values for the reference gene *actb1* averaged 25.4 ± 2.1. The detection of *actb1* failed in 4 out of the 390 (0.7%) of the swabs analysed. Although basal gene expression levels of *c3*, *lysG*, and *IgMsec* were measured in skin swabs from control fish independent of the holding temperature, their detection by qPCR failed in a high proportion of the swabs tested (25% of invalid test for *c3*, 9.75% for *IgMsec*, and 8.25% for *lysG*). Basal levels of *IgZ2* were only detected in control fish held at 17 °C and within that group, only 15% of the swabs were analysed.

In challenged fish, failure to detect the targeted genes in skin swab samples was seen in 36% of the swabs for the detection of *c3*, 8.5% for *IgMsec*, and 4.4% for *lysG*. *IgZ2* was only detected in 3.8% of the swabs analysed showing high Ct values.

### 2.4. Mucosal Shedding of KHV Takes Place Independently of the Primary Temperature of Infection

**Figure 3:**
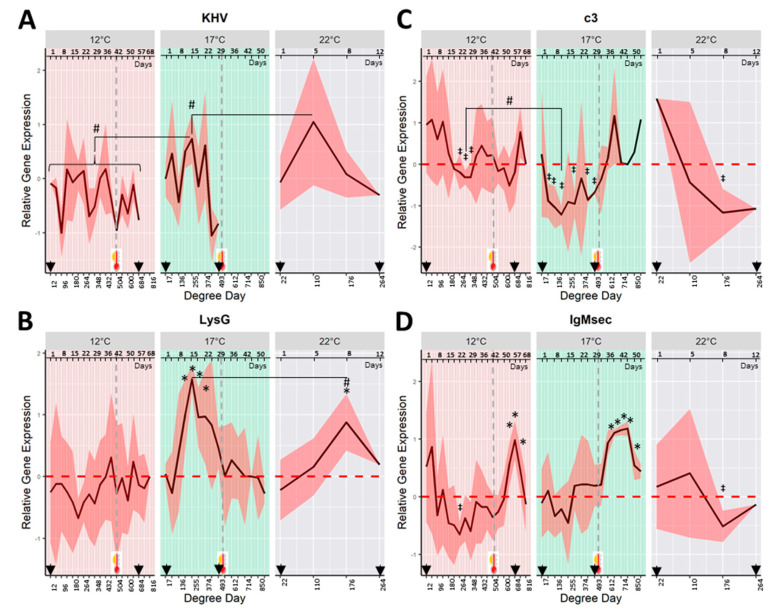
Mean (continuous black line) of the relative gene expression (fold change) over time in common carp skin swabs exposed to koi herpesvirus (KHV). Sampling days expressed as degree days (DD) to compare groups at different temperatures (12, 17, and 22 °C). A continuous line was used instead of columns to show gene expression patterns over time. Shadow represents 95% confidence intervals around the mean. (**A**) mRNA KHV *orf90* (viral shedding); (**B**) *lysozyme g* (*lysG*); (**C**) *complement c3-h1* (*c3*); (**D**) *immunoglobulin M heavy chain secretory protein* (*IgMsec*). Arrowheads indicate the window of KHV shedding. (*, ‡) represents gene expression significantly higher (*) or lower (‡) from the control group when *p* ≤ 0.05. (#) Represents significant differences among treatments. The vertical black dotted line and thermometer denote a change in the temperature (increase to 22 °C heat stress) at ~500 DD, which corresponded to 42 and 30 days after the challenge for fish at 12 °C and 17 °C respectively. Note for Figure 3 and Figure 4: The number of fish sampled each day at 12 °C = 12. The number of fish sampled at 17 and 22 °C overtime decreased due to mortalities. At 17 °C, from 17 to 204 DD = 12 fish each sampling day; from 255 to 342 DD = 10 fish; from 342 to 374 DD = 9 fish; from 374 to 442 DD = 8 fish; from 442 to 612 DD = 6; from 612 to 850 DD = 5; from 850 to 918 = 4. At 22 °C, at 22 and 110 DD = 12 fish; at 176 DD = 9 fish; and at 264 DD = 3 fish.

KHV *orf90* transcripts were detected in skin swabs from fish exposed to KHV at 22 °C from day 1 to day 12 (last moribund fish). The highest viral shedding was measured after 5 days (110 DD) post-challenge, coinciding with the observations of severe KHVD clinical signs (Figure 3A).

Symptomatic viral shedding was also detected in mucus samples of fish challenged at 17 °C from day 1 to 29 (17–493 DD). Higher viral replication (shedding episode peaks) was measured on the sampling days 5, 15, and 22 (85, 255, and 374 DD). There were no detectable levels of viral shedding after heat stress (>500 DD). The relative amount of virus shed during the highest shedding episode for fish held at 22 °C (peak at 110 DD) was significantly higher (*p* < 0.05) than the peak measured at 17 °C (peak at 255 DD).

Fish exposed at 12 °C showed asymptomatic viral shedding from day 1 to 57 (12–684 DD), which included viral replication measured after heat stress (500 DD). The relative amount of viral RNA transcripts in skin mucus was lower (*p* < 0.05) for fish held at 12 °C than at 17 or 22 °C. KHV was not detected in the skin of control fish.

### 2.5. Mucosal Parameters Trends in KHV Infection by Temperature Treatment

Despite the fact that both challenged and control fish showed a high standard deviation of the gene expression of *lysG*, *c3*, *IgM*, and *IgZ2* on mucus samples during the sampling dates, different patterns of gene expression were observed between challenged and control fish and temperature treatments.

In fish exposed to KHV at 22 and 17 °C, the gene expression of the host *lysG* showed a marked pattern of up-regulation during the viral shedding (Figure 3B). *LysG* was significantly up-regulated (*p* < 0.05) at day 8 (176 DD) in fish held a 22 °C, and from days 12 to 22 (204–374 DD) in fish at 17 °C when compared with their respective control fish. The highest average *lysG* up-regulation was observed on day 8 for fish at 22 °C, and on day 15 for those at 17 °C (176 and 255 DD respectively). At both temperatures, the maximum *lysG* up-regulation occurred 3 days (for fish at 22 °C) and 10 days (for fish at 17 °C) after the first peak of KHV shedding. The peak of the *lysG* gene expression for fish held at 17 °C was significantly higher (*p* < 0.05) than the peak at 22 °C. *LysG* gene was not differently expressed in fish held at 12 °C.

Over time, the *c3* gene expression of fish challenged at 22 and 17 °C showed a marked pattern of down-regulation coinciding with the window of viral shedding (Figure 3C). The observed down-regulation was significantly different from control fish at day 8 (176 DD) for fish challenged at 22 °C, and several sampling points from 5 to 29 days (85 to 493 DD) for fish held at 17 °C. An increase in the *c3* gene expression, to levels similar to negative control fish, was measured in those survivors experiencing temperature increase from 17 to 22 °C.

A significantly down-regulated pattern of *c3* was also observed at 12 °C from days 22 to 29 (264–348 DD). *C3* down-regulation at 17 °C was significantly lower than that observed at 12 °C (*p* < 0.05) (*c3* average of relative gene expression at the lowest peak was −0.42 ± 0.23 for fish held at 12 °C at 312 DD vs. −1.25 ± 0.24 and −1.16 ± 0.55 for 17 and 22 °C at 204 and 110 DD respectively).

The relative gene expression of *IgMsec* of fish at 22 °C showed a peak of down-regulation on day 8 (176 DD) (Figure 3D). For fish held at 17 °C, overall, changes in *IgMsec* gene expression were not significantly different from control fish. However, immediately after the heat stress at 500 DD, the gene expression of *IgMsec* experienced a significant (*p* < 0.05) up-regulation from days 36 to 50 (612–850 DD). Fish held at 12 °C showed a pattern of down-regulation of *IgMsec* gene expression with a minimum at day 22 (264 DD). As observed at 17 °C, the gene expression of *IgMsec* showed a significant up-regulation after increasing the temperature from 12 to 22 °C from days 54 to 62 (648–744 DD).

**Figure 4:**
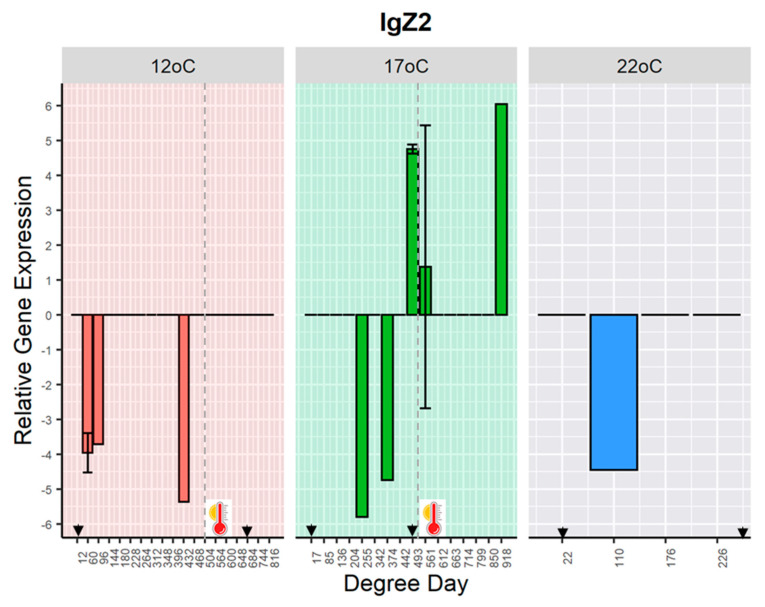
Normalised gene expression of *immunoglobulin Z2 heavy chain* (*IgZ2*) over time in common carp skin swabs exposed to koi herpesvirus (KHV). Arrowheads indicate the window of KHV. The vertical black dotted line and thermometer denote a change in the temperate (heat stress) at ~500 DD (42 and 30 days after the challenge for fish at 12 °C and 17 °C respectively).

The gene expression of *IgZ2* was detected sporadically in some challenged fish held at 12 °C, at days 5, 8 and 36 (60, 96 and 432 DD); at 17 °C, at days 15, 20, 22, 33, and 54 (255, 342, 374, 493, 561 and 918 DD); and 22 °C at day 5 (110 DD) post-challenge (Figure 4). The detection of *IgZ2* transcripts occurred in some samples where *IgM* mRNA was either low expressed or not detected (Figure S3).

### 2.6. Gene Expression Correlations over Time

**Figure 5:**
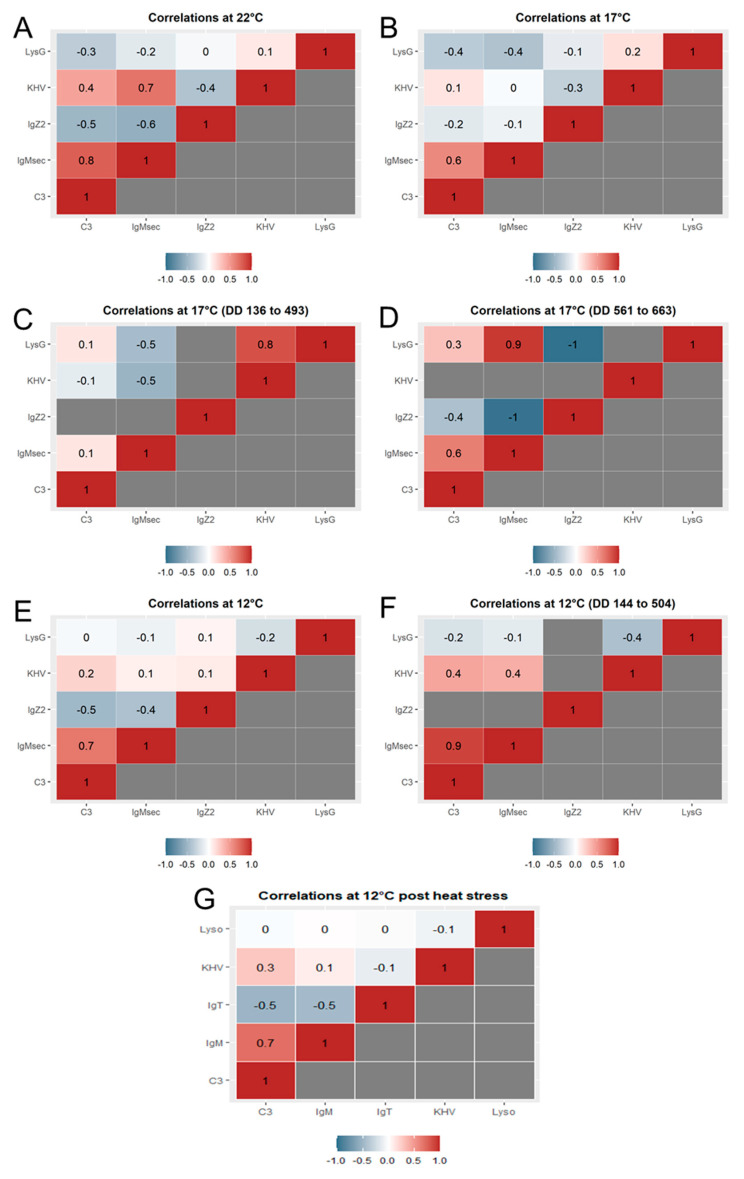
Pairwise correlation heat maps of the relative gene expression of *complement c3-h1* (*c3*), *lysozyme g* (*lysG*), *immunoglobulin M heavy chain secretory protein* (*IgMsec*), and *immunoglobulin Z2 heavy chain* (*IgZ2*) in skin swabs taken from common carp exposed to koi herpesvirus (KHV) at either 22 °C (**A**), 17 °C (**B**–**D**), or 12 °C (**E**–**G**). Numbers indicate Pearson’s correlation coefficients. DD: degree day.

At 22 °C, positive correlations (both genes up-regulated or down-regulated following the same pattern) were observed between *IgMsec* and *c3* (Pearson’s *r*-value of 0.8) and KHV *orf90* and *IgMsec* (Pearson’s *r*-value of 0.7) (Figure 5A).

At 17 °C, the correlation between *IgMsec* and *c3* was also observed (Pearson’s *r*-value of 0.6) over the full duration of the study (Figure 5B), and when interrogating in more detail, over the later (post heat stress) but not earlier time series (Figure 5C,D). When the pairwise comparisons were conducted using the narrowed time series coinciding with the viral replication after the first shedding episode, a significant positive correlation (Pearson’s *r*-value of 0.8) between KHV replication and *lysG* was seen from day 8 to 29 (Figure 5C). After the heat stress, the gene expression of *IgMsec* and *lysG* showed a strong positive correlation (Pearson’s *r*-value of 0.9) from days 33 to 39 (Figure 5D). A strong negative correlation (Pearson’s r value of −1) was obtained between *IgZ2* and *lysG* and *IgZ2* and *IgMsec* from days 33 to 39.

Similarly, at 12 °C, a positive correlation (Pearson’s *r*-value of 0.7) between *IgMsec* and *c3* was observed (Figure 5E–G). In contrast to the observations at 17 °C, this pairwise correlation was statistically stronger before the heat stress (Pearson’s *r*-value of 0.9) from days 12 to 42 (Figure 5F).

### 2.7. Comparison of the Mucosal Immunity between Survivors and Moribund Fish Held at 17 °C

**Figure 6:**
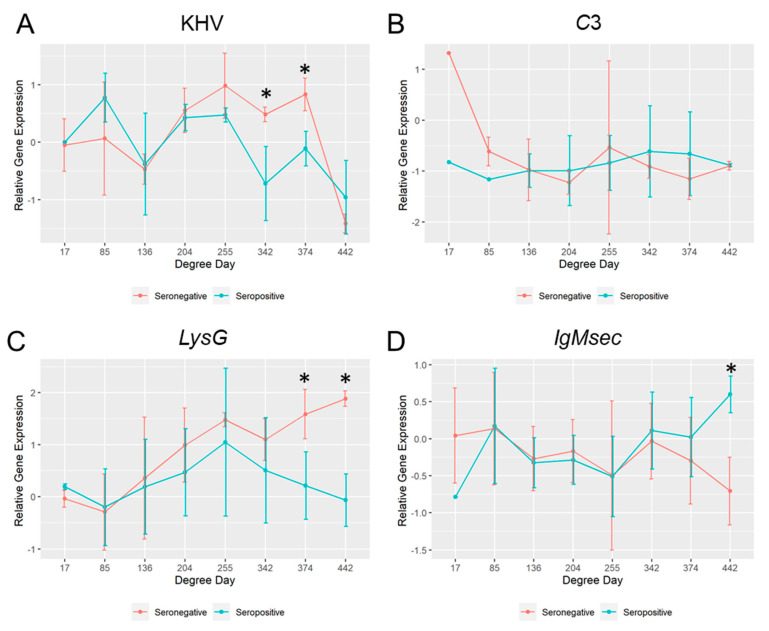
Relative gene expression (fold change) over time in skin swabs from common carp exposed to koi herpesvirus (KHV) held at 17 °C that died before the heat stress (seronegative group) vs. survivors (seropositive). (**A**) KHV *orf90* (viral shedding); (**B**); *complement c3-h1* (*c3*); (**C**) *Lysozyme g* (*lysG*); (**D**) *immunoglobulin M heavy chain secretory protein* (*IgMsec*). (*) Denotes gene expression significantly different (*p* ≤ 0.05) among groups.

Among the fish challenged at 17 °C, fish that died before the heat stress (<500 DD) were seronegative. When the mucosal parameters of those seronegative fish were compared with fish that survived for more than 500 DD (seropositive fish), differences between groups were observed (Figure 6). In the seronegative group, the KHV viral replication and the host *lysG* gene expression were significantly higher at 342 and 374 DD (sampling day 20 and 22) and 374 and 442 DD (sampling day 22 and 26) respectively. However, *IgMsec* was significantly higher at 442 DD (26 days post-challenge) in the seropositive group. No significant differences in the *c3* gene expression were observed among them, apart from the first sampling point at 1 day after the challenge (17 DD), where the observed mRNA basal levels were different.

### 2.8. KHV Carrier Status

Brain samples were analysed to determine wherever KHV DNA was present in seropositive fish at the end of the challenge (~900 DD). All survivors, independently of the initial temperature of exposure to the virus, tested positive for KHV. Viral load in 50 mg of brain tissue ranged from 6.5 × 10^7^ to 1.3 × 10^7^ copy numbers of the KHV *orf90* gene (Ct values in 2.5 µL of DNA: 24.6 ± 0.7 for fish exposed at 17 °C and 27.8 ± 0.3 for fish at 12 °C). The results are summarised in Table 1.

## 3. Discussion

A surveillance programme conducted in 2009 in the UK showed that fish that have been exposed to the pathogen (KHV seropositive) were widespread and prevalent in fisheries distributed throughout England and Wales, with a high number of seropositive sites (circa 1500) compared to the number of observed clinical case sites per year (Circa 15) [3,38]. An examination of historical KHVD incidences in those seropositive sites suggested that many outbreaks were related to new introductions of naive cohorts of fish [38]. Mathematical modelling of KHV epidemiology predicted that introducing new cohorts at the end of autumn (coinciding with decreasing temperatures) in positive KHV sites might suppress the mortality associated with KHV and lead to a high prevalence of immunity in the population [11]. Typically, in river catchments in southern England, the average freshwater temperature of stream water fluctuates between 9 to 16 °C over a year [39], while in fisheries, it averages between 4 °C to 21 °C [40]. In the present study, common carp were exposed to KHV mimicking three different scenarios: 12 °C (cold season) as non-permissive temperature, 17 °C (warm season) as UK typical KHV-permissive temperature, and 22 °C (heat stress) as optimal KHV temperature.

Consistent with previously published work [9,10,15], carp exposed to KHV at 17 °C and 22 °C showed typical KHV clinical signs, reaching cumulative mortalities of 60% (17 °C before heat stress), 80% (17 °C after heat stress) and 100% (22 °C). As shown previously, fish that did not elicit an antibody response at permissive temperatures (17 and 22 °C) did not survive the initial exposure [9], and all survivors became persistently infected with the virus regardless of the initial holding temperature (12, 17 or 22 °C) [8,15,41]. Within permissive temperatures, despite a higher level of antibody being observed after increasing the temperature from 17 °C to 22 °C, further mortalities were recorded in some of those seropositive fish, which could be associated with viral recurrences [15]. However, in the present study, viral shedding in the skin mucus was not detected after heat stress in those seroconverted fish. At a non-permissive temperature of 12 °C, all fish survived the initial infection and subsequent heat stress at 22 °C, displaying only mild KHV clinical signs, which supports the hypothesis behind natural immunization for KHV by water temperature control [11]. In a previous study, carp infected at 11 °C did not show signs of disease and the gene expression of the viral *thymidine kinase* was absent or greatly restricted even though viral DNA was measured in skin mucus samples [42]. Similarly, in the present study, lower levels of asymptomatic viral shedding were measured at 12 °C when compared with symptomatic shedding episodes measured at 17 °C and 22 °C, however, the episodes of viral shedding at low temperature occurred over a prolonged period (57 days for fish at 12 °C vs. 29 days for fish at 17 °C). This wider window of viral shedding might be in part explained due to the failure of seroconversion before the heat stress. Antigen processing and recognition are relatively temperature-independent, however, the interaction between T and B cells and posterior release of antibodies by plasma cells are considered to be temperature-dependent [43]. Other steps in the immune response might also be temperature-dependent, which could explain the lack or delay of seroconversion, hence the long window of KHV asymptomatic shedding after the primary exposure. Previous work using sheep red blood cells as antigen proved that carp could mount a delayed humoral response at temperatures below 12 °C at 49 days (588 DD), followed by a strong anamnestic response after a second exposure to a higher temperature (>20 °C) [44], and that a prolonged interval between primary and secondary injection leads to a higher antibody secondary response [45]. While in the current study seroconversion and 100% survival were measured after the heat stress in fish exposed at 12 °C, other studies reported mortality rates of 45%–50% after inducing viral reactivation in infected carp held at 11 °C and 12 °C for 28 and 38 days (308 and 456 DD) respectively; lamentably, seroconversion was not addressed in those studies [15,37]. Thus, it seems that the length of the holding period at non-permissive temperature could partially explain disparities observed in the viral reactivation and survival after the heat stress.

To comprehend the dynamics of viral shedding and host response at the mucosal compartment, the gene expression of four selected genes that play a role in mucosal immunity (*lysG*, *c3*, *IgMsec*, and *IgZ2*) was measured at the three different holding temperatures. As expected, Taqman qPCR Ct values obtained from non-invasive skin swabs were higher than those typically measured from skin samples (i.e., *actb1* Ct average in skin swabs was 25.4 ± 2.1 vs. 15.6 ± 0.7 in skin tissues [34]). Ct values obtained in this study for the housekeeping gene were similar to those reported previously from buccal swab samples [46], however, the Taqman qPCR detection of the targeted genes failed in a proportion of the swabs analysed. While skin swabs might not be suitable when analysing low-level gene expression, for which mRNA enrichment or further RNA purification would be required, the non-lethal sampling method allowed for a considerable reduction in the number of animals used and allowed a close follow up of the response of each animal to the viral infection over time.

Although it is not known what triggers KHV re-activation following temperature increases, high levels of cortisol as a result of temperature stress have been related to the viral re-activation in koi carp [16]. Changes in plasma lysozyme have been widely used as a stress indicator in fish [47]. In the present study, significant changes in the *lysG* gene regulation were not observed during the viral shedding in those fish kept at low temperature, probably due to the inhibitory effect of colder temperature as shown for lysozyme levels in serum [30]. However, a significant up-regulation of *lysG* transcripts during the window of viral shedding was observed at higher temperatures. It was suggested that the up-regulation of the *c-type lysozyme* (*lysC*) in the skin of common carp exposed to KHV could be a response of secondary bacterial infection [34]. Lysozyme in fish could also be acting as an acute-phase protein involved in the overall alarm response to pathogen infection [23]. However, the strong positive correlation of *lysG* up-regulation with the viral shedding observed in the present study could also suggest a specific antiviral role of lysozyme in the KHV infection. Increased up-regulation of lysozyme in the serum of mirror carp was measured during KHV infections [48], and in rohu (*Labeo rohita*) after induction with poly I:C, a dsRNA viral analogue [49]. Lysozyme plays an important role in the non-specific immune system; its antiviral function has been described in higher vertebrates, yet further studies are required to understand its role during viral infections in teleost fish [26].

The expression of lysozyme typically activates the complement system and phagocytes [26]. In teleost fish, the complement systems modulate adaptive immune responses, pro-inflammatory reactions, the elimination of apoptotic and necrotic cells, and the destruction of pathogens [19]. In the present study, despite the strong up-regulation of *lysG* in mucus samples, a significant down-regulation of *c3* mRNA transcripts in the skin was seen independent of the initial holding water temperature during the window of viral shedding, which returned to basal expression levels after the heat stress coinciding with the clearance of the virus from the skin. Down-regulation of the complement system by mammalian herpesviruses has been described involving multiple adaptation strategies, such as the viral encoding of genes to interact with complement proteins (glycoproteins C), which can bind to C3 and its activation products; glycoproteins gE and gI, which form a heterodimeric complex and function as an IgG Fc receptor facilitating immune evasion by promoting ‘antibody bipolar bridging’; kidnapping complement receptors (CD46) and complement regulators (CD55, CD59) from the host cells; and encoding homologs of regulators of complement activation to elude the complement system (for review see [50]). C3 is a central component of the complement system and serves as a marker of both the classical, lectin, and alternative pathways. KHV infection in common carp, as well as Cyprinid Herpesvirus 2 (CyHV-2) infection in gibel carp (*Carassius auratus gibelio*), induced up-regulation of *c3* transcription in internal organs (liver, spleen, and kidney) [51,52], but not at the mucosal site (gills) even though a strong up-regulation of *bf/c2* transcripts, involved in the classical and alternative complement pathways, was observed [51]. Hence, the present study might suggest a subversion of the teleost complement system by KHV at the mucosal compartment during viral shedding, though the mechanisms involved in the host complement evasion are still to be elucidated.

Viral complement evasion not only impairs innate immunity but also adaptive immunity (antibody-dependent complement activation). Indeed, a significant correlation among *IgMsec* and *c3* was measured independent of the water holding temperature, both showing a similar pattern of down-regulation during the viral shedding window, followed by a pattern of up-regulation after the heat stress, likely coinciding with seroconversion and clearance of KHV from the mucosal surface. Moreover, when the mucosal response of fish challenged at a permissive temperature of 17 °C was assessed, *IgMsec* was up-regulated in the survivors coinciding with the end of the shedding episode. Thus, the results suggest that a weak immunoglobulin induction might contribute to the long term mucosal shedding, similar to that described for murid herpesvirus 4 (MuHV-4) and herpes simplex virus 1 (HSV-1) in a mouse model [53], and that high concentration of *IgMsec* in the skin of common carp prevents further viral replication, similar to that observed for IgG concentration in mucus secretions during herpesvirus infections in humans [54].

In teleost fish, genes coding for the human homologues IgM and IgD, and the unique immunoglobulin in fish, IgZ subclasses 1 and 2 (in cyprinids) or IgT (in salmonids), have been characterised so far [55]. Due to the TCRα/TCRδ loci arrangement in fish, the expression of IgM and IgZ are mutually exclusive on B-cells [20,55]. Indeed, in the present study, IgZ2 transcripts were only detected sporadically in those skin swabs where *IgMsec* mRNA was absent or low expressed, and this exclusion was corroborated by a strong negative correlation among *IgZ2* and *IgMsec* observed at 17 °C after the heat stress. Thus, the results showed that *IgM*, and not *IgZ2*, is mainly involved in the host response to KHV infection at the skin-associated lymphoid tissue.

Due to the establishment of a life-long infection by KHV and subsequent episodes of viral reactivation and shedding, it is vital to understand the effect of the temperature on the viral infection and host response to design strategies to reduce infectivity at the mucosal sites and consequently transmission. The present study showed that the host response to KHV infection at the mucosal site was similar at the three different temperatures analysed, except for lysozyme which was inhibited at low temperature. The gene expression patterns suggested that KHV replication might subvert the complement system, which coupled with a weak antibody response at the mucosal site, favours long periods of viral shedding. The fact that a high survival rate after heat stress is recorded when the primary infection takes place during non-permissible temperatures might allow for fish movements into KHV-positive sites, where water temperature management and interventions to minimise viral shedding are possible.

## 4. Materials and Methods

### 4.1. Ethics Statement

Animal procedures were approved by the Animal Welfare and Ethical Review Body (AWERB) at the Cefas Weymouth Laboratory and conducted in compliance with the Animals (Scientific Procedures) Act 1986 Amendment Regulations 2012.

### 4.2. Viral Inoculum Preparation

A European strain of KHV, K250, isolated from a KHV outbreak in the UK in 2007, was propagated in the common carp brain (CCB) derived cell line (ECACC 10072802) at 20 °C in EMEM media (Sigma, Gillingham, UK) supplemented with 2 mM Glutamine, 1% non-essential amino acids (Sigma, Gillingham, UK), 2% Foetal Bovine Serum (FBS), and 10 mM HEPES (Sigma, Gillingham, UK) [56].

The supernatant of cells showing cytopathic effects was harvested, clarified by centrifugation at 4000× *g* for 15 min, and stored at 4 °C.

To determine the viral dose, the copy number of the KHV *orf90* gene was quantified in a Taqman qPCR test previously described [8]. Viral nucleocapsid was extracted from the clarified supernatant using the EZ1 Virus Mini Kit and the EZ1 extraction robot (Qiagen, Manchester, UK) following the manufacturer’s instructions. To generate a standard curve, a fragment of 1450 bp of the KHV *orf90* gene containing the qPCR region was cloned into the pGem-T Easy plasmid vector (Promega, Southampton, UK) as described before [57]. The template (dsDNA) copy number was calculated using a QuantiFluor dsDNA kit in a Quantus fluorimeter (Promega, Southampton, UK), and a plasmid dilution series, from 10^6^ to 1 copy, was generated.

### 4.3. KHV Challenge and Sampling Regime

Thirty-six specific pathogen-free (SPF) common carp, weighing ~40 g, were split into three groups of 12 fish each and acclimated at 22, 17, and 12 °C respectively. Ten fish per group were then exposed to 15 mL of virus stock in 30 L of tank water on an aerated static bath for 4 h, equivalent to a final viral load of ~2.4 × 10^6^ copies (Ct value of 14.9 ± 0.04) of KHV isolate K250. Following the bath challenge, fish were held in individual tanks at their corresponding temperature with a flow rate of ~0.4 L min^−1^. Two fish for each temperature were mock challenged with culture media as controls. Fish were held for 500 degree days (DD), equivalent to 22 days for fish held at 22 °C, 29 days for fish held at 17 °C, and 42 days for fish held at 12 °C, after which the animals were subjected to heat stress by increasing the temperature to 22 °C over 5 days for fish kept at 12 °C and over 3 days for those at 17 °C following previous challenge models for KHV reactivation [15,16]. Fish were monitored for a further 22 days after the heat stress.

To determine the levels of anti-KHV antibody on sera, a non-lethal blood sample was taken under anaesthesia from all the survivors at 500 DD, followed by a second blood sample at 900 DD, after which fish were terminated. Blood samples were also taken from all moribund fish before termination.

For all fish, repetitive skin swabs (Figure S1), using Isohelix DNA/RNA Buccal Swabs (Merck Life Sciences, Southampton, UK), were taken 1 day after the challenge and then every ~4 days until the end of the challenge for gene expression analysis and viral shedding. Swabs were immediately frozen at −80 °C until further analysis.

At the end of the challenge (~900 DD), brain sections were sampled and stored in ethanol at −20 °C for measuring viral latency/persistence in survivors.

### 4.4. RNA Extraction from Skin Swabs and Taqman qPCR Analysis

A total of 390 skin swabs collected over the challenge were soaked on 100 µL of RLT lysis buffer (Qiagen, Manchester, UK) and vortexed to dislodge the cells and cellular components attached to the swab matrix, after which the swab was removed and the total RNA extracted from the cell suspension using an EZ1 RNA Tissue Mini Kit and an EZ1 extraction robot (Qiagen, Manchester, UK), with final elution buffer of 50 µL of buffer following the manufacturer’s instructions.

Total RNA was reverse transcribed at 37 °C for 1 h in a 20 µL reaction containing 4 µL of the extracted RNA; 200 U of M-MLV RT, M-MLV RT 5× reaction buffer (250 mM Tris-HCl, pH 8.3; 375 mM KCl; 15 mM MgCl2; 50 mM DTT); 1 mM dNTP mix; 500 ng of random primers; and 25 units RNasin^®^ Ribonuclease Inhibitor (Promega, UK).

Taqman qPCR assays were performed for each sample in duplicate in a master mix containing 2 µL of the cDNA, 500 nM of each primer, and 250 nM of probe labelled with 6-FAM 5′ and 3′ BHQ1, in a total volume of 20 µL by using the Taqman^®^ Universal Master Mix II with UNG (Applied Biosystem, Southampton, UK). PCR reactions were performed on a StepOne Real-Time PCR apparatus with V2.3 software (Applied Biosystem, Southampton, UK) with 40 cycles of qPCR amplification and fluorescence detection as recommended by the manufacturer. Molecular grade water was used as a negative control for each master mix.

Taqman qPCR assays were de novo designed to analysing the gene expression of four common carp immune-related genes from the skin swabs: the *complement* c3-h1 (*c3*) [40]; *lysozyme g* (*lysG*) [41]; *immunoglobulin M heavy chain secretory protein* (*IgMsec*) [42]; and *immunoglobulin Z2 heavy chain* (*IgZ2*) [43]. *Actin beta 1* (*actb1*) was used as a reference gene for normalization purposes [34] (Table 2).

The viral shedding was measured from the same RNA extracted from skin swabs using a previously published Taqman qPCR test assay for the detection of the KHV *orf90* gene [8].

### 4.5. Gene Expression and Statistical Analysis

Serial tenfold dilutions of a cDNA sample were used to generate standard curves to determine each primer set efficiency, giving slope values close to −3.3 (Figure S2).

Inter run calibrated normalized relative quantities (CNRQ) in the gene expression, equivalent to the fold change method (2^∆∆*C*t^) [58], were calculated using qbase+ version 3.2 software (Biogazelle, UK) [59]. Normalised gene expression data were visualised through graphical plots generated in R, version 3.6.1 [60].

An analysis of the variance (ANOVA) test was conducted to determine significant differences in the gene expression (when *p* value < 0.05) at each temperature treatment between challenged and control fish, and between challenged fish groups at different temperatures.

To study correlations among genes and between genes and the viral replication in a time series, pairwise relationships were calculated using Pearson’s correlation coefficient (*r* values) in R, version 3.6.1. The correlation analysis was assayed in two ways: Firstly, pairwise relationships between the normalised gene expression values at each temperature using all the sampling points before and after heat stress, and secondly, pairwise relationships using a narrowed time series to address gene correlations either before or after the heat stress.

### 4.6. Antibody ELISA Tests

To measure the levels of anti-KHV antibody in blood samples, an indirect ELISA test for the detection of anti-KHV antibody in common carp serum was conducted as described elsewhere [3]. Unheparinised blood samples were allowed to clot overnight at 4 °C and, following centrifugation at 1500× *g* for 10 min, sera were stored at −20 °C for subsequent ELISA tests. Dilutions of sera at 1:50 and 1:100 were tested. For coating the ELISA plates, KHV was propagated in CCB cells as described above and the clarified supernatant ultracentrifuged at 100,000× *g* for 1 h at 4 °C in Optima 523 XPN-100 ultracentrifuge (Beckman Coulter, Southampton, UK) to pellet the viral particles. Viral protein concentration was measured with a BCA protein assay kit (Abcam, Cambridge, UK), adjusted to 10 µg mL^−1^, and used as a capture antigen in the ELISA plates. The optical density (OD) was measured at 450 nm in a microplate reader Spark 10 M using Magellan™ data analysis software (Tecan, Theale, UK). Controls were included in each ELISA plate consisting of wells without sera and wells without the anti-common carp IgM monoclonal antibody (Aquatic Diagnostics, Stirling, UK).

The collected data were analysed using Student’s *t*-test to determine significant differences among groups when *p* < 0.05.

### 4.7. KHV Latency/Persistency in Survivors

To determine if surviving fish still harboured viral DNA, which could inform their status as KHV carriers, brain samples were analysed at the end of the challenge at approximately ~900 DD (54 and 68 days for survivors exposed at 17 and 12 °C respectively). Briefly, brain sections (~0.05 g) were homogenised 1:10 weight/volume in lysis buffer (Promega, Southampton, UK) and digested with proteinase K (4 mg mL^−1^) at 56 °C for 10 min. Viral nucleocapsid was then extracted using the RSC Viral Total Nucleic Acid Purification Kit in a Maxwell^®^ RSC Instrument (Promega, Southampton, UK). KHV *orf90* gene was detected using the diagnostic Taqman qPCR described above, and the Ct values extrapolated to the number of copies of the viral genome using the KHV diagnostic standard curve described above.

## Figures and Tables

**Table 1:** Table showing summary results for common carp exposed to KHV either non-permissive (12 °C) or permissive temperatures (17 and 22 °C). For each temperature treatment before and after heat stress (temperature increase to 22 °C); survival rates; seroconversion at 500 and 900 degree days; the window of KHV shedding; the gene expression patterns of *lysozyme g* (*lysG*), *complement c3-h1* (*c3*), *immunoglobulin M heavy chain secretory protein* (*IgMsec*), *immunoglobulin Z2 heavy chain* (*IgZ2*); and pairwise correlations among them are shown. +ve: positive; -ve: negative; OD: ELISA test optical density; NA: not applicable.

		12 °C	17 °C	22 °C
Before heat stress	Survival	100%	40%	0%
	Seroconversion 500 DD	Negative	Morts: Negative Survivors: Positive, OD = 0.31 ± 0.06	Negative
	KHV shedding	From 1 to 41 days Peaks: 12, 22, 36 days	From 1 to 29 days Peaks: 5, 15, 22 days	From 1 to 12 days Peak: 5 days
	*lysG*	No different from controls	Peak of up-regulation	Peak of up-regulation
	*c3*	Down-regulation pattern	Down-regulation pattern	Down-regulation pattern
	*IgMsec*	Down-regulation pattern	No different from controls	Down-regulation pattern
	*IgZ2*	Sporadic detection	Sporadic detection	Sporadic detection
	Gene correlations	*IgMsec _c3*: +ve	*IgMsec _c3*: +ve KHV_ *lysG*: +ve	*IgMsec _c3*: +ve KHV_ *IgMsec*: +ve
Post heat stress (22 °C)	Survival	100%	20%	NA
	Seroconversion 900 DD	Positive, OD = 0.45 ± 0.12	Morts +ve, OD = 0.35 ± 0.04 Survivors +ve, OD = 0.51 ± 0.08	NA
	KHV shedding	From 41 to 57, Peak at 42 and 50	Negative	NA
	*lysG*	No different from controls	No different from controls	NA
	*c3*	No different from controls	No different from controls	NA
	*IgMsec*	Up-regulation pattern	Up-regulation pattern	NA
	*IgZ2*	Not detected	Sporadic detection	NA
	Gene correlations	*IgMsec _c3*: +ve	*IgMsec _c3*: +ve *IgMsec*_*lysG*: +ve *IgZ2_lysG*: -ve *IgZ2*_*IgMsec*: -ve	NA
	KHV carrier status	100%	100%	NA

**Table 2:** Summary of the common carp *Cyprinus carpio* genes and nucleotide sequences of the primers and probes used for the Taqman qPCR assays: *beta-actin* (*actb1*); *complement c3-h1* (*c3*); *lysozyme g* (*lysG*); *immunoglobulin M heavy chain secretory protein* (*IgMsec*); and *immunoglobulin heavy chain Z2* (*IgZ2*).

Gene	GenBank No.	Forward Primer 5′-3′	Reverse Primer 5′-3′	Probe 6-Fam 5′- 3′ MGB
Reference gene				
*actb1*	JQ619774.1	CACCATGTACCCTGGCATTG	GAGGGAGCAAGGGAGGTGAT	TGACCGTATGCAGAAGG
Complement				
*c3*	AB016211.1	TGGGTGATGTACGTGGCAAA	GCATGGCAATCACGACAAAA	TGCCGATGCCTCTC
Antimicrobial				
*lysG*	AB084624.1	ACCCGCAGGCAATGGTT	CGGAGTGTGGGAGCGTTT	TGGCCTTATGCAGGTTG
Immunoglobulins				
*IgMsec*	MH352354.1	GTGCCGAAGCCTGGAAGA	GCCTTGTGCACGAACTCACA	CAAACCAGCAAAGTT
*IgZ2*	AB598368.1	CATCACCTGCCCCGTAAGAT	TGACACTTGTAACGGAGCATTTG	TGGCAACATGAAGGAT

## Supplementary Materials

The following are available online at https://www.mdpi.com/1422-0067/21/22/8482/s1, Figure S1: Example of common carp skin swabbing procedure; Figure S2: Taqman qPCR efficiency curves. Positive cDNA dilutions 1:10. 1:100 and 1:1000; Figure S3. Relative gene expression over time in common carp skin swabs exposed to koi herpesvirus (KHV).
