# Supplementary material for: Seroconversion and Skin Mucosal Parameters during Koi Herpesvirus Shedding in Common Carp, Cyprinus carpio
Source: Int J Mol Sci. 2020 Nov 11;21(22):8482. doi: 10.3390/ijms21228482 (PMC7696817; doi:10.3390/ijms21228482)
Supplement: Supplementary file 1 [file ijms-21-08482-s001.pdf]

Supplemental material

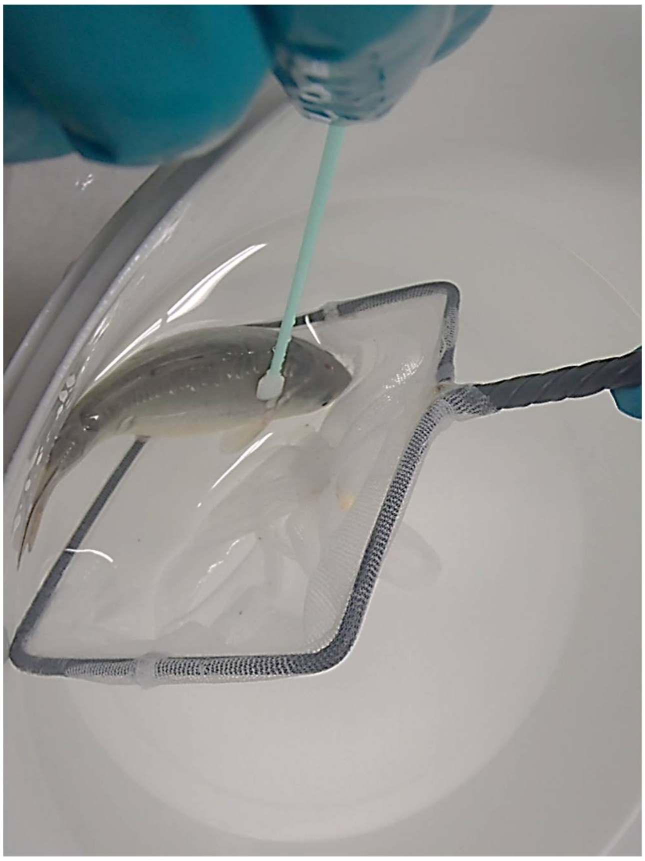

Figure S1. Example of common carp skin swabbing procedure.

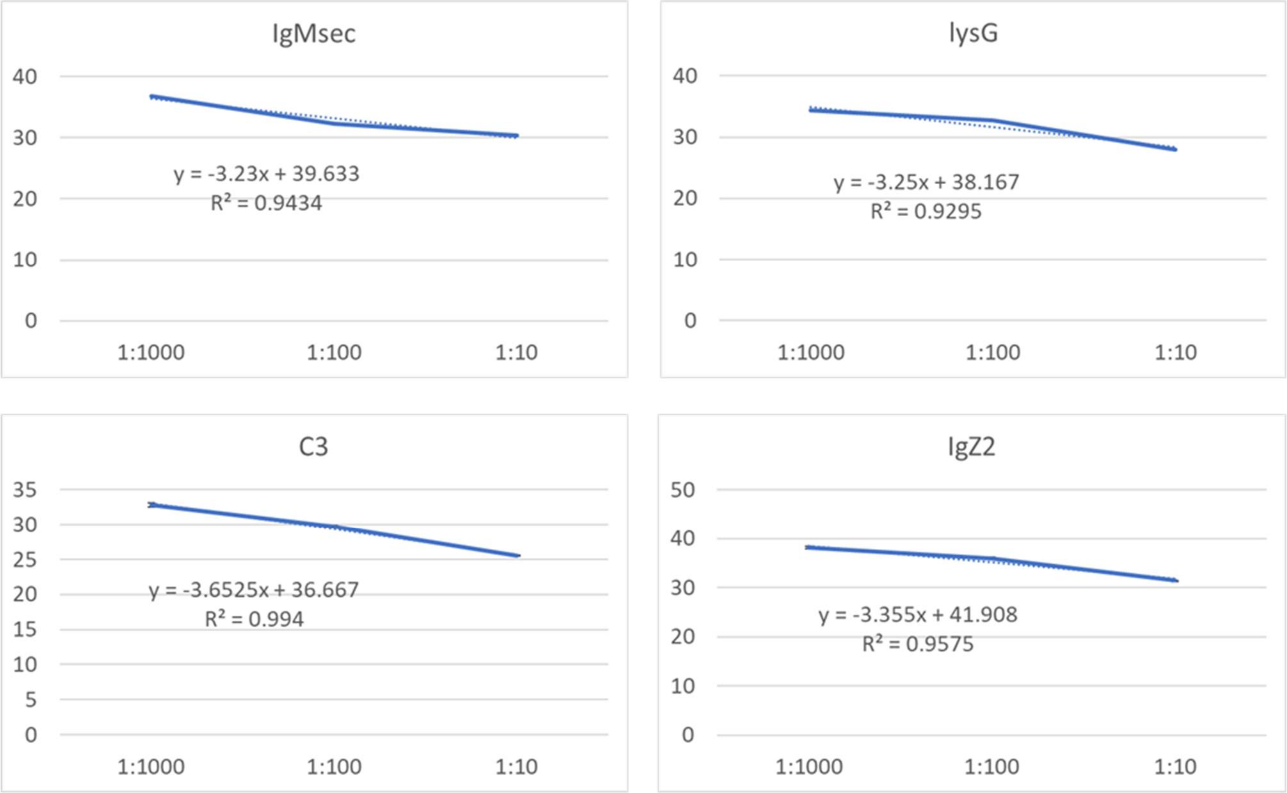

Figure S2. Taqman qPCR efficiency curves. Positive cDNA dilutions 1:10. 1:100 and 1:1000.

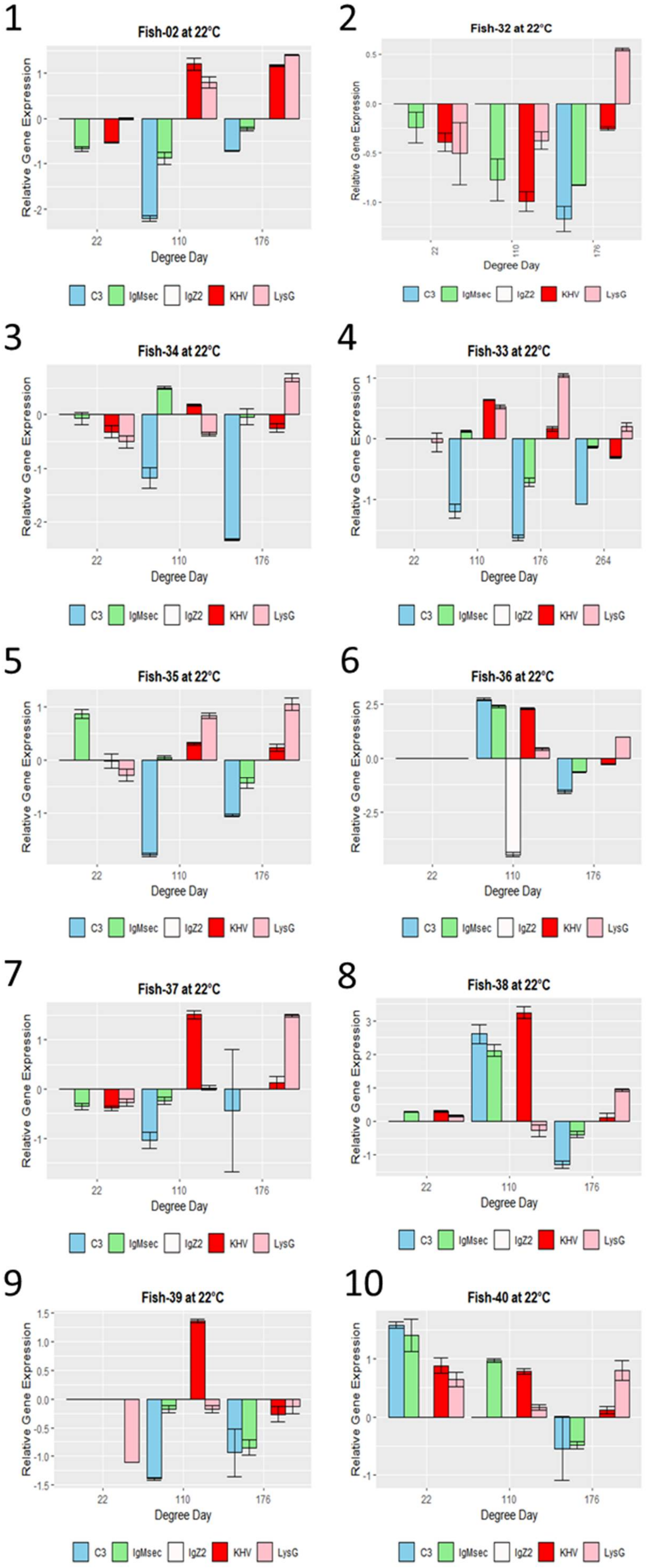
1
Fish-02 at 22°C
2
Fish-32 at 22°C
3
Fish-34 at 22°C
4
Fish-33 at 22°C
5
Fish-35 at 22°C
6
Fish-36 at 22°C
7
Fish-37 at 22°C
8
Fish-38 at 22°C
9
Fish-39 at 22°C
10
Fish-40 at 22°C
Relative Gene Expression
Degree Day
22
110
176
264
C3
IgMsec
IgZ2
KHV
LysG

11

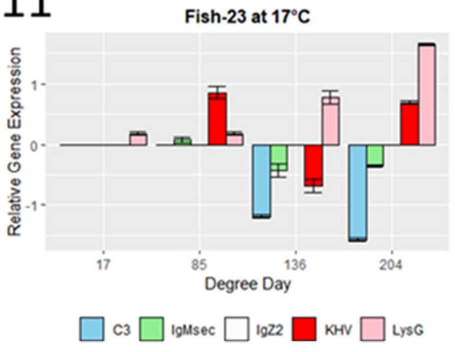

12

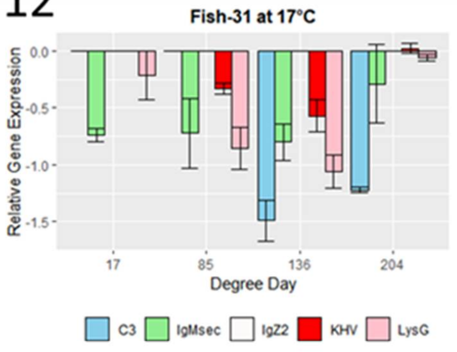

13

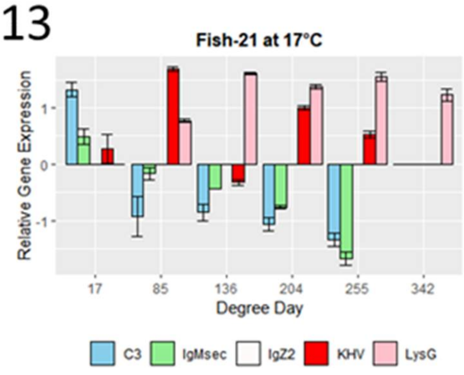

14

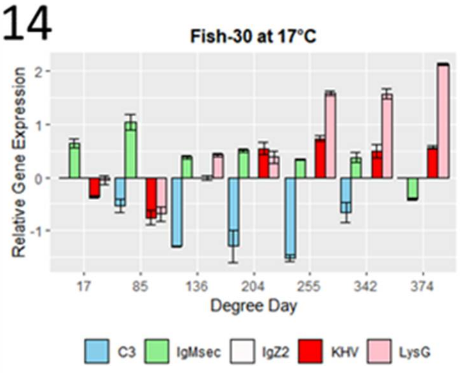

15

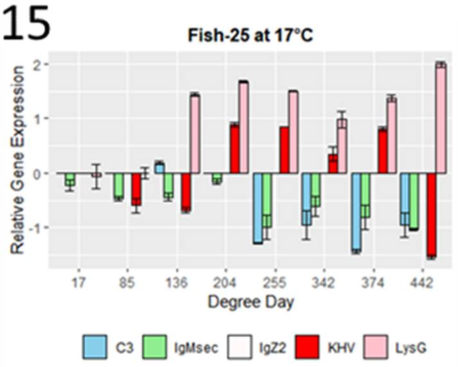

16

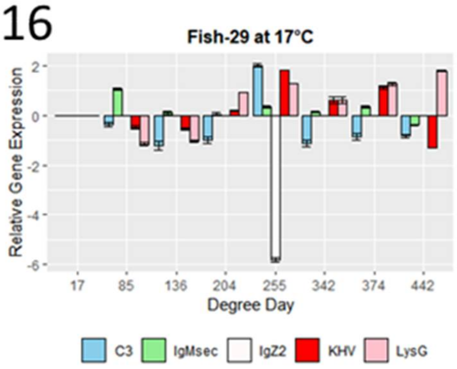

17

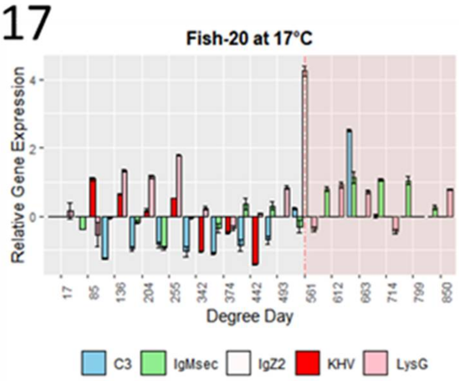

18

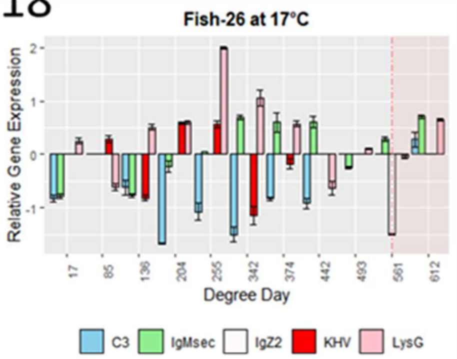

19

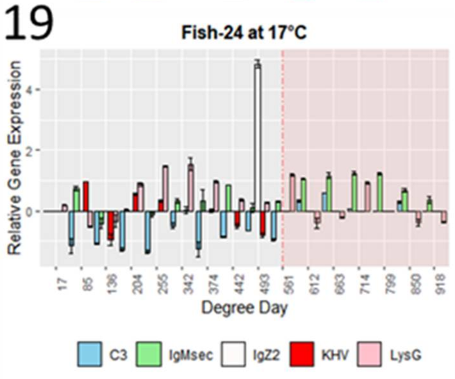

20

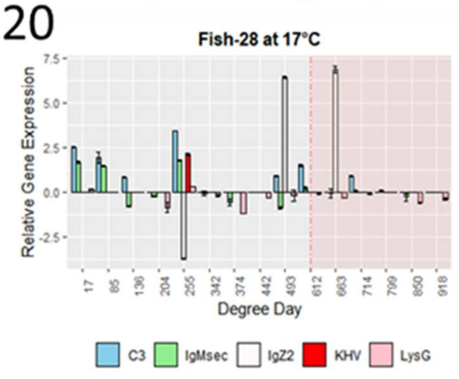

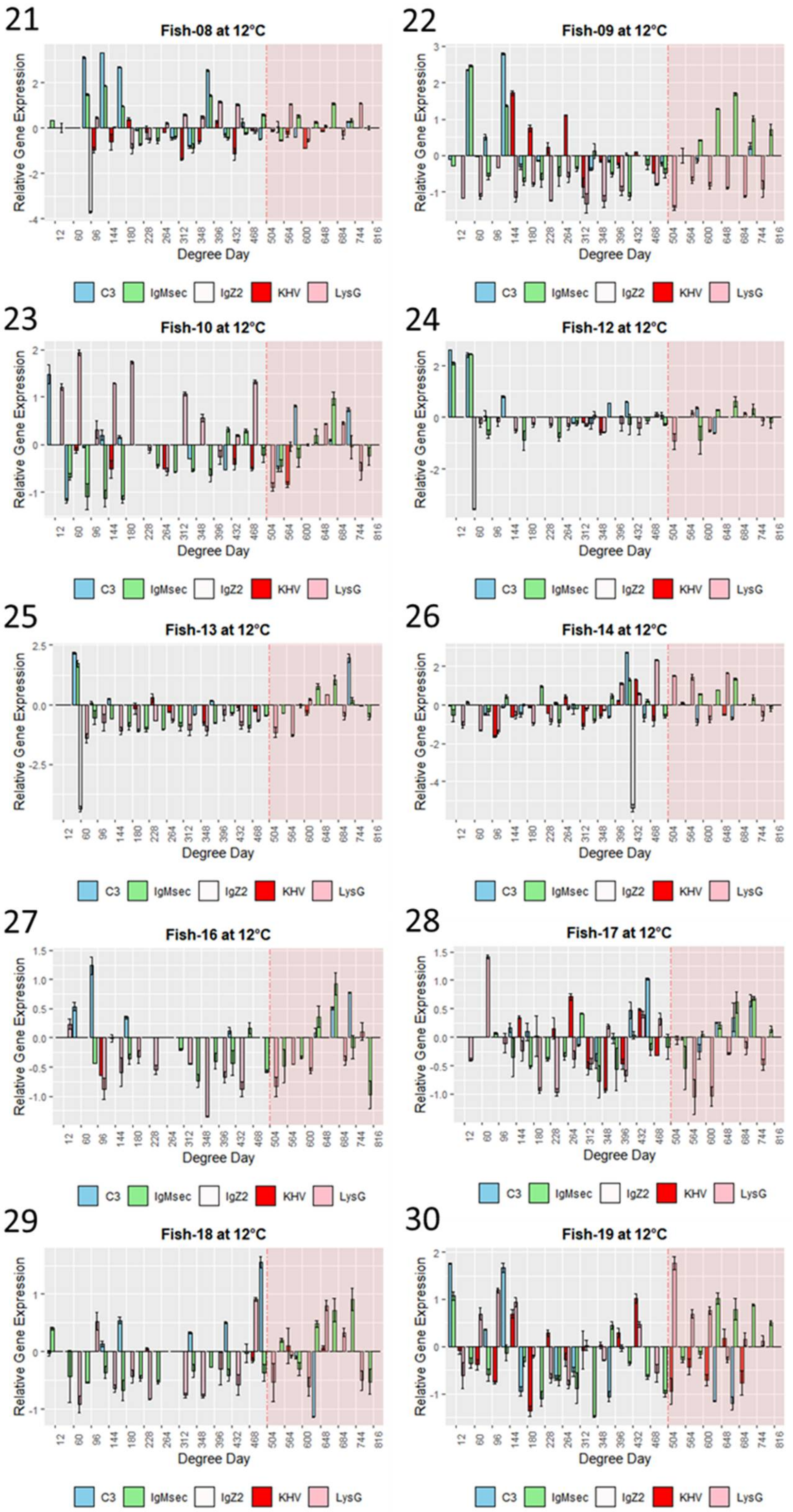

Figure S3. Relative gene expression over time in common carp skin swabs exposed to koi herpesvirus (KHV). Sampling days were expressed as degree days (DD) to compare groups at different temperatures (12, 17, and 22 °C). *C3*: *complement c3-h1*; *IgMsec*: *immunoglobulin M heavy chain secretory protein*; *IgZ2*: *immunoglobulin Z subclass 2 heavy chain*; KHV: KHV *orf90*; *lysG*: *Lysozyme g*. Graphs from 1 to 10: fish infected at 22°C; graphs from 11 to 20: fish infected at 17°C and survivors exposed to heat stress at 500 DD; graphs from 21 to 30: fish infected at 12°C and survivors exposed to heat stress at 500 DD. Heat stress indicates an increase in temperature to 22°C.
